# Teratoma Models

**Published:** 1980-04

**Authors:** M. J. Evans


### TERATOMA MODELS

M. J. EVANS

*From the Department of Genetics, University of Cambridge*

THERE HAS LONG been a suspicion that neoplasia might be in part a disease of differentiation. The juxtaposition of these two aspects of cellular behaviour is nowhere else seen so dramatically as in teratocarcinomas, which are malignant tumours, the stem cells of which not only proliferate rapidly but also differentiate into a wide variety of types of differentiated cell which are themselves essentially non-malignant. The biology of these tumours in mice has been elucidated largely by Stevens (1967). Pierce (1967) and his collaborators laid the foundations of the exploration of the cell biology involved. In subsequent years it has been shown that the stem cells (embryonal carcinoma cells) may be established as clonal tissue-culture cell lines. They will differentiate fully as well *in vitro* as *in vivo* (Martin & Evans, 1975) and some lines have been shown capable of integration with an early mouse embryo and to give rise to substantial parts of the subsequent normal mouse (Papaioannou *et al.*, 1978).

The growth and differentiation of teratocarcinoma cells *in vitro* is thus established as a tractable system (Martin, 1975) and exploration of its cell biology and molecular biology has proceeded on a number of fronts. Here the relationship between embryonal carcinoma cells and their putative normal counterparts in the embryo is considered. As teratocarcinomas may be induced experimentally by the ectopic implantation of early embryos (up to 7½ days old or of germinal ridges containing undifferentiated germ cells) pluripotential early embryonic cells or primordial germ cells would seem to be likely candidates for the origin of teratocarcinomas. The expression of cell-surface antigens recognised by monoclonal antisera, and the major patterns of protein synthesis seen by two-dimensional electrophoresis, have been compared for embryonal carcinoma cells and various embryonic cell types.

These results suggest that choices are made in cell determination in which only one daughter line emerges at a time. The stem line continues in the same state until it subsequently changes in itself (Evans *et al.*, 1979). The most likely homologues of embryonal carcinoma cells are the cells of the inner cell mass of the early 5-day embryo.

REFERENCES

EVANS, M. J., LOVELL-BADGE, R. H., STERN, P. L. & STINNAKIE, M. G. (1979) *Cell Lineages Stem Cells and Cell Determination.* p. 115.

MARTIN, G. R. (1975) *Cell*, **5**, 229.

MARTIN, G. R. & EVANS, M. J. (1975) *Cell*, **6**, 467.

PAPAIOANNOU, V., GARDNER, R. L., McBURNEY, M. W., BABINET, C. & EVANS, M. J. (1978) *J. Embryol. Exp. Morphol.*, **44**, 93.

PIERCE, G. B. (1967) *Current Topics Dev. Biol.*, **2**, 223.

STEVENS, L. C. (1967) *Adv. Morph.*, **6**, 1.


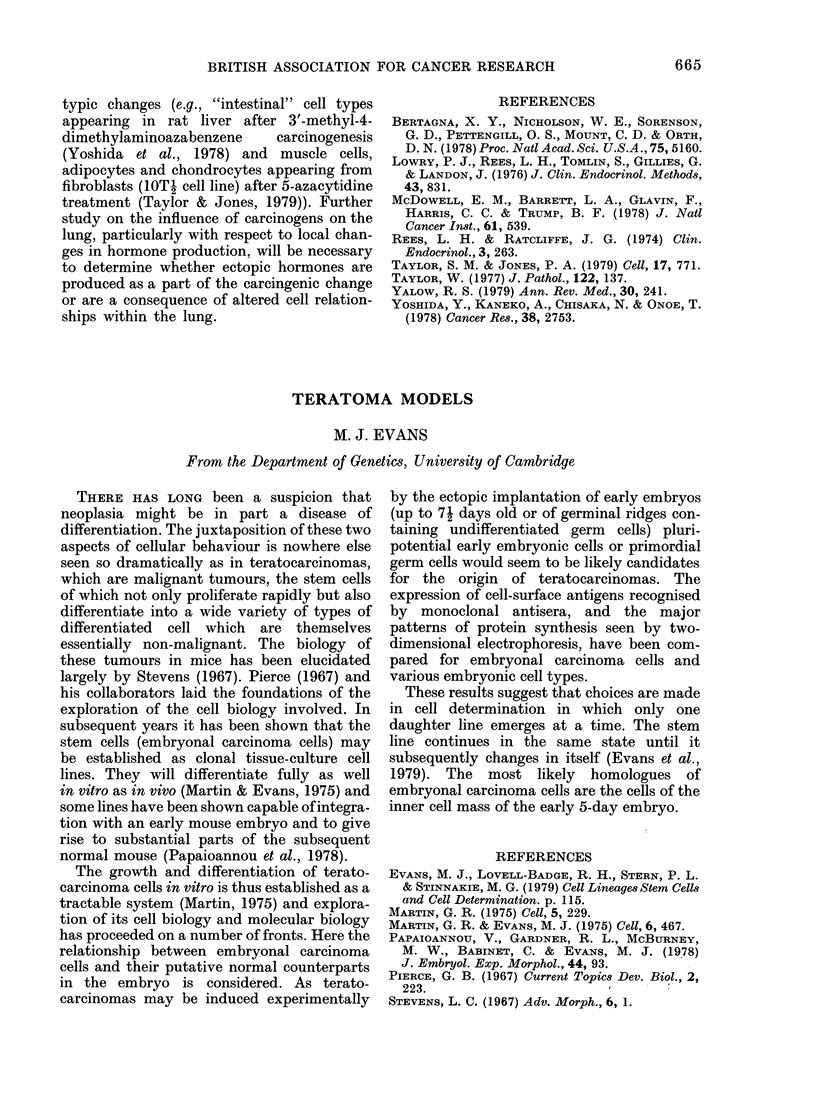


## References

[OCR_00071] Martin G. R. (1975). Teratocarcinomas as a model system for the study of embryogenesis and neoplasia.. Cell.

[OCR_00075] Papaioannou V. E., Gardner R. L., McBurney M. W., Babinet C., Evans M. J. (1978). Participation of cultured teratocarcinoma cells in mouse embryogenesis.. J Embryol Exp Morphol.

